# Irreversible specialization for speech perception in early international adoptees

**DOI:** 10.1093/cercor/bhab447

**Published:** 2021-12-25

**Authors:** Gunnar Norrman, Emanuel Bylund, Guillaume Thierry

**Affiliations:** Centre for Research on Bilingualism, Department of Swedish Language and Multilingualism, Stockholm University, SE-106 91, Stockholm, Sweden; Centre for Research on Bilingualism, Department of Swedish Language and Multilingualism, Stockholm University, SE-106 91, Stockholm, Sweden; Department of General Linguistics, Stellenbosch University, 7602 Matieland, Stellenbosch, South Africa; School of Human and Behavioural Sciences, Bangor University, Bangor, Gwynedd, LL57 2DG, United Kingdom; Faculty of English, Adam Mickiewicz University, Collegium Heliodori, ul. Grunwaldzka 6, 60-780, Poznań, Poland

**Keywords:** critical period, event-related brain potentials, international adoption, language acquisition, mismatch negativity

## Abstract

In early childhood, the human brain goes through a period of tuning to native speech sounds but retains remarkable flexibility, allowing the learning of new languages throughout life. However, little is known about the stability over time of early neural specialization for speech and its influence on the formation of novel language representations. Here, we provide evidence that early international adoptees, who lose contact with their native language environment after adoption, retain enhanced sensitivity to a native lexical tone contrast more than 15 years after being adopted to Sweden from China, in the absence of any pretest familiarization with the stimuli. Changes in oscillatory brain activity showed how adoptees resort to inhibiting the processing of defunct phonological representations, rather than forgetting or replacing them with new ones. Furthermore, neurophysiological responses to native and nonnative contrasts were not negatively correlated, suggesting that native language retention does not interfere with the acquisition of adoptive phonology acquisition. These results suggest that early language experience provides strikingly resilient specialization for speech which is compensated for through inhibitory control mechanisms as learning conditions change later in life.

## Introduction

Language development in early childhood proceeds through a series of nested critical or optimal periods during which specific aspects of language ability are established (Werker and Tees 2005; Werker and Hensch 2015; Reh et al. 2020). The first year of life in particular is crucial for tuning neural and behavioral sensitivity to native speech sounds (Kuhl et al. 1992; Cheour et al. 1998; Kuhl 2004), which later provide a foundation for higher order linguistic skills (Tsao et al. 2004; Rivera-Gaxiola et al. 2005). One way to gauge the importance of language development during this period is to study sequential bilinguals, who have acquired a second language after initial exposure to one language. However, such an approach is confounded by changes in linguistic processing arising due to the co-existence of two sets of language representations. A solution is to study individuals who receive normal language input in early life but undergo a radical change in their language environment after initial sensitivity has been established. Such is the case of international adoptees, individuals who are born to a particular language environment, but who have transferred, literally overnight, to a different environment, never to be significantly exposed to their native language again.

Previous studies have suggested that neuroplasticity is extended in the case of adoptees due to a loss of birth language traces, enabling the later establishment of nativelike patterns of language processing (Pallier et al. 2003; Ventureyra et al. 2004). But recent investigations have shown that early development can leave long-term traces of a neural commitment to the native language (Pierce et al. 2014, 2015). It is unclear whether such traces—referred to here as specialization—become dormant due to lack of maintenance (Tees and Werker 1984; Bowers et al. 2009; Hyltenstam et al. 2009; Singh et al. 2011; Pierce et al. 2014) or compete with brain systems supporting adoptive language acquisition and processing (Pallier et al. 2003; Hernandez et al. 2005). In addition, the extent to which the constraints established in a toddler can be overcome later in life remains largely unknown (Friederici et al. 2002; Uylings 2006).

To address these important developmental questions, we studied the most sensitive and selective neurophysiological index of phonological discrimination available, the mismatch negativity (MMN), in healthy adult participants who were adopted from China to Sweden at a mean age of 18 ± 11 months. The MMN is a negative modulation of auditory event-related potentials maximal over frontocentral regions of the scalp between 150 and 300 ms after stimulus onset (Näätänen and Picton 1987). It is unconsciously and spontaneously elicited in the human auditory cortex and surrounding regions, when an infrequent stimulus (deviant) is detected as perceptually salient among a series of frequent ones (standards; Näätänen 1995). We presented early Chinese–Swedish adoptees with phonological contrasts marking either a native Mandarin Chinese lexical tone variation (high-flat vs. high-rising) or an adoptive Swedish vowel contrast (/--u / vs.  /y /). Crucially, neither contrast was phonologically relevant in the other language, the two lexical tones of Chinese being phonologically indistinguishable in Swedish (Riad 2014) and the two vowels of Swedish undifferentiated in Chinese (Duanmu 2007). We further tested one group of native speakers of Chinese with minimal experience of Swedish and one group of native speakers of Swedish with no experience of Chinese to assess adoptees in relation to typical native language development outcomes.

We hypothesized that early language experience would render the adoptees more sensitive to a native tone contrast relative to the adoptive vowel contrast, despite having no conscious recollection of being exposed to Mandarin Chinese in early childhood. This pattern was expected in the Chinese natives also, while Swedish natives were expected to exhibit the inverse response pattern. In addition to comparing differences in MMN amplitude modulation for the two conditions within the three participant groups, we computed variations in spectral frequency power and coherence over time. This analysis allowed us to distinguish between functional brain networks with different oscillatory pattern signatures (Makeig et al. 2004).

We found that international adoptees retain enhanced pre-attentive sensitivity to a native Chinese contrast they have not been exposed to for over 15 years compared with an adoptive language contrast. This sensitivity pattern was obtained in the absence of Chinese re-exposure of familiarization before testing, and remarkably similar to that found in Chinese controls, but not to the native Swedish controls, suggesting long-term retention of sensitivity to that contrast. Oscillatory brain responses furthermore suggest inhibitory control involvement in response to the defunct tone contrast in the adoptees, indicating compensatory mechanisms.

## Materials and Methods

### Participants

Three groups with different language learning backgrounds were included in the study: adults adopted from China to Sweden as children (*N* = 19, 18 females, age = 20.2 ± 1.8 years), Chinese native speakers (*N* = 22, 10 females, age = 23.0 ± 3.4 years), and Swedish native speakers (*N* = 22, 16 females, age = 22.5 ± 3.0 years). The adoptees had arrived in Sweden in early childhood (age at adoption = 18.5 ± 11.3 months; range 5–48 months). While some reported having had brief contact with Chinese (e.g., through short visits to China during childhood) or receiving mother tongue instruction during the first years of school, none of them could speak Chinese, had any knowledge of the language, nor had received any extended exposure to Chinese since adoption. The Chinese natives were exchange students, tested within 2 months of arrival in Sweden, none having any prior knowledge of Swedish. They grew up primarily in the northern parts of mainland China and were fluent in Mandarin Chinese. The Swedish natives were born in Sweden, grew up in Stockholm, and had no prior experience with Chinese. All participants were recruited in Stockholm, Sweden, through advertisements in local newspapers, online participant recruitment services, and word-of-mouth. Participants were paid a nominal amount for their participation (about $7 per hour). Due to restrictions in the availability of international adoptees and Chinese control participants, it was not possible to perfectly match participant groups for age, gender, and handedness. While only right-handed participants were selected for the control groups, four participants in the adopted group were left-handed. This was controlled for by adding age, gender, and handedness as covariates in the statistical models (see Analyses). The method and procedure complied with the requirement of the declaration of Helsinki was approved by the regional ethical review board of Stockholm prior to testing, and all participants signed informed consent forms before participating in the study.

### Stimuli

We compared a Chinese lexical tone contrast (high flat vs. high rising) and a Swedish vowel contrast (high front rounded vs. high central rounded), both of which only existed in one of the two languages. In Mandarin Chinese, every syllable can be realized with four different tones (high, high-rising, falling-rising, and falling), each of which marks a distinction in meaning between words. While the tone inventory may differ between dialects, we used a level-rising contrast that is present in all major Chinese dialects. Swedish has up to 18 distinct vowels (distinguished by both formant structure and duration; Riad 2014), whereas Chinese has five basic vowels (Duanmu 2007). This study used the Swedish contrast between a high front rounded /y/ and a high central rounded vowel /--u /. The Chinese pitch envelopes were modeled on a recording of a native Chinese speaker and were combined with Swedish formant structures, to create four unique stimuli. The stimuli were created using a vocal tract synthesizer (Vocal Tract Lab; Peter Birkholtz, www.vocaltractlab.de). They were 250 ms in duration and normalized in amplitude to 65 dB (see Supplementary Fig. 1).

### Procedure

The task consisted of an oddball paradigm in which frequent stimuli (Standards) are presented sequentially, pseudorandomly interspersed with infrequent stimuli (Deviants). We used a dual Deviant variant of the paradigm (two different Deviants within each block) and there were at least two Standards between two consecutive Deviants. The average number of Standards between two Deviants was 4 ± 2.5. Each individual stimulus served as a Standard once (*N* = 800 per block, 80%), as a vowel Deviant once (*N* = 100 per block, 10%), and as a lexical tone Deviant once (*N* = 100 per block, 10%). Stimulus onset asynchrony was randomly jittered between 700 and 900 ms. Stimuli were counterbalanced across four experimental blocks and block order was counterbalanced between participants.

Participants were seated in a sound-attenuated room and watched a silent comedy movie presented on a 4-inch screen (iPod touch) placed 50–80 cm in front of them, in order to minimize eye movements. Auditory stimuli were presented using E-Prime and delivered using insert tube earphones with a flat frequency response (Etymotic ER-1). Each block lasted about 15 min, with a brief pause between blocks. The experiment lasted about 60 min in total.

### E‌EG Recording and Preprocessing

EEG was recorded from a 32-channel Ag/AgCl high-impedance system (BioSemi ActiveTwo system with active electrodes; BioSemi) with electrodes mounted in a cap and covering the following locations, conforming to the 10–20 convention (Sharbrough et al. 1991): FP1, AF3, F7, F3, FC1, FC5, T7, C3, CP1, CP5, P7, P3, Pz, PO3, O1, Oz, O2, PO4, P4, P8, CP6, CP2, C4, T8, FC6, FC2, F4, F8, AF4, FP2, Fz, and Cz. Electrooculograms were recorded from electrodes placed above and below the left eye, and from positions lateral to the left and right eyes. Additional electrodes were placed on the left and right mastoids and on the tip of the nose for offline referencing.

Data preprocessing was performed using the Fieldtrip Toolkit (Oostenveld et al. 2011) in Matlab (version 2020a, The MathWorks). Raw data were down-sampled to one-fourth (512 Hz) of the original sample rate prior to processing. Continuous recordings were high-pass filtered at 0.1 Hz (cutoff −6 dB, order 8448, transition bandwidth 0.2 Hz) using a one-pass zero-phase Hamming windowed sinc FIR filter, and then low-pass filtered at 40 Hz (cutoff −6 dB, order 564, transition bandwidth 3 Hz). Motor artifacts and segments with excessive noise were removed manually from the continuous data, which was then split into epochs. Eye blinks were identified using independent components analysis (ICA, using the *runica* function implemented in Fieldtrip) of individual trials and were mathematically corrected. Only the ICA component most likely associated with vertical eye movements was discarded from each participant’s data. Any remaining trials where the amplitude exceeded ±75 V within −0.2 to 0.5 s (i.e., the epoch duration) relative to stimulus onset were removed from the data. Three datasets (one in each group) had less than 60 valid trials for at least one deviant in one of the blocks and were removed from further analysis. ERPs were computed by taking the average of all baseline corrected trials (−0.2 s relative to stimulus onset) for each condition combined across the four experimental blocks.

The time–frequency representation of the cleaned and epoched data was computed from single trials using a 0.5-s Hanning taper applied in steps of 50 ms from −0.5 to 1 s relative to stimulus onset (implemented in the Fieldtrip Toolbox, *ft_freqanalysis* function with the *mtmconvol* method). Frequencies ranged between 0.5 and 20 Hz with a resolution of 0.5 Hz. The Fourier representation was converted into spectral power by averaging the squared absolute values of the complex Fourier spectra for each stimulus type (Standard, tone Deviant, vowel Deviant) across the four blocks for each participant. Individual epochs were normalized to prestimulus average spectral power and log transformed (10^*^log10) to yield relative power change in decibels over time. Intertrial phase coherence was computed from the Fourier representation using the following formula:

$$ \mathrm{ITC}\left(f,t\right)=\frac{1}{N}\sum_{i=1}^h\frac{F_k\left(f,t\right)}{\mid{F}_k\left(f,t\right)\mid } $$

where | | represents complex norm (Delorme and Makeig 2004). Phase coherence for the standard stimuli was subtracted from each deviant condition, and the resulting difference was used in the statistical analysis.

### Data Analyses

Amplitudes for deviants and standards immediately preceding a deviant were averaged and the deviant-minus-standard difference was computed for each condition. Inspection of the nose-referenced plots showed inverted polarity at the mastoids compared with the vertex electrode, as would be expected for MMN responses generated in the superior temporal cortex (Näätänen and Picton 1987). Due to the higher degree of noise at the nose electrode, an average mastoid reference was used for statistical analysis and plotting. Peak latency was measured individually as the largest negative peak in the deviant-minus-standard difference wave at Fz in the MMN time window.

Statistical comparison was conducted by means of cluster-based permutation tests (Maris and Oostenveld 2007; see also Sassenhagen and Draschkow 2019) implemented in Fieldtrip. After significant clusters were identified, data within each cluster were randomly exchanged between conditions (Montecarlo simulation) and the significance level for each cluster was assessed in relation to the permutation distribution. This procedure provides a robust nonparametric test of significance while controlling for family-wise error rate. The *Montecarlo* method used 1000 iterations and two-tailed *t*-tests (alpha level = 0.05). This procedure was used to identify the electrodes and time windows in which conditions deviated in the time and frequency domains.

Group differences were assessed using linear mixed-model regression (implemented using the fitlme function in Matlab). For each model, categorical factors were effect coded (−1, 1) as follows: group (adopted, Chinese, Swedish) and condition (tone, vowel). Main effects and interactions were included in the model as predictors. Individual variation in responses was controlled for by including by-participant random intercepts. Since the groups differed in terms of age and gender, these factors were included as covariates in the models. Some participants reported as left-handed and the laterality quotient derived from the Edinburgh handedness inventory (Oldfield 1971) was included as a binary covariate with values above zero treated as right-handed and values below zero treated as left-handed.

## Results

### Adult International Adoptees Retain Native Tone Contrast Sensitivity

**Figure 1:**
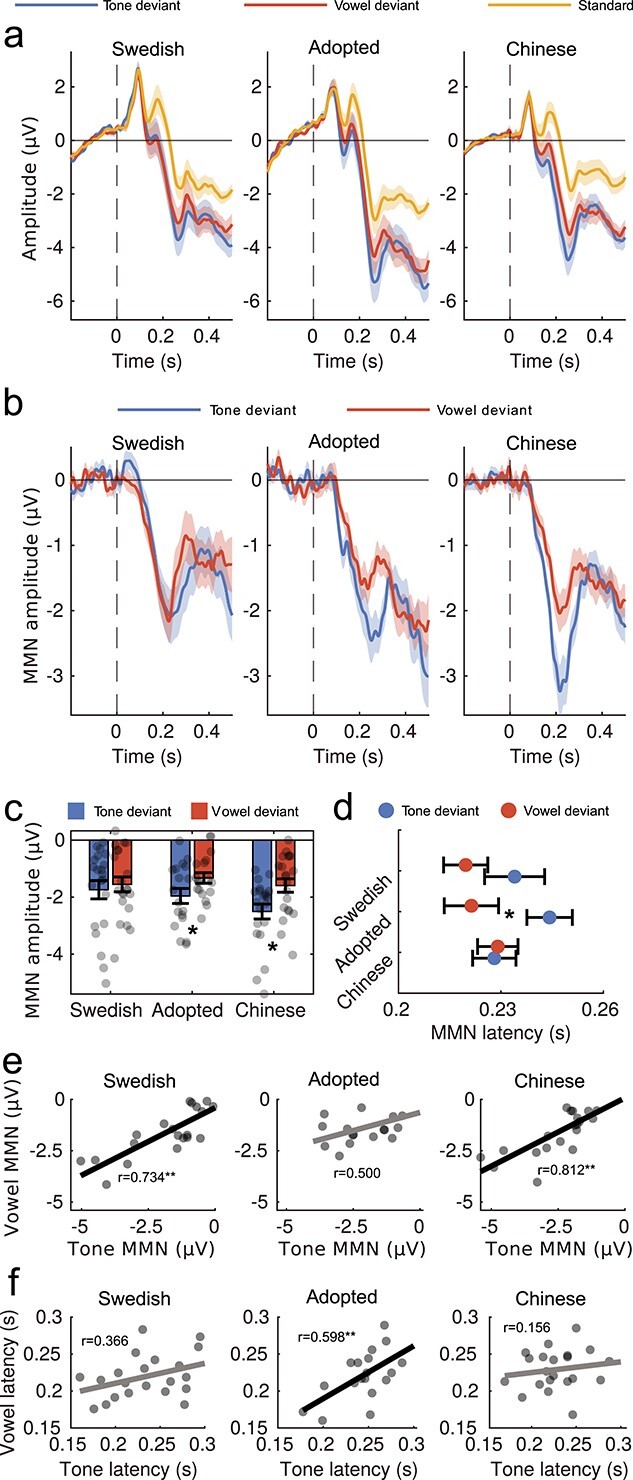
MMN results. (*a*) Event-related brain potentials elicited by standards, tone, and vowel deviants in the three participant groups; (*b*) MMN modulation (deviant—standard) in each of the three participant groups; (*c*) bar plot of mean MMN amplitude measured between 0.15 and 0.3 s; jittered dots depict individual participant means; (*d*) MMN peak latency for all three groups in the two deviant conditions (tone, vowel), measured as the largest negative peak between 0.15–0.3 s; (*e*) regression plots of mean MMN amplitude modulation for the tone contrast against the vowel contrast and Pearson correlation scores; (*f*) regression plots of mean MMN peak latency (^*^P < 0.05, ^^*^^*^^P < 0.01). All measures are taken from representative electrode Fz. Error bars and shading depict s.e.m.

The two phonological contrasts used elicited a significant N1 peak modulation in all three participant groups, irrespective of whether the contrast was specific to Chinese (lexical tone variation) or Swedish (vowel contrast; Fig. 1*a*), observed as a negative cluster peaking at fronto-central sites starting around 100 ms. Differences in amplitude between responses to deviant and standard stimuli, the MMN, had a similar amplitude and morphology for tones and vowels in Swedish natives, but international adoptees and Chinese natives showed increased responses to the tone contrast relative to the vowel contrast (Fig. 1*b*). Cluster-based permutation tests on the deviant-minus-standard difference wave showed increased negativity for the tone relative to the vowel contrast in Chinese natives (*t*_sum_ = −1895.28, *P* = 0.001, between 0.13 and 0.3 s) and adoptees (*t*_sum_ = −850.86, *P* = 0.007, 0.23–0.32 s), but not in Swedish natives. All analyses were conducted at representative electrode Fz averaged between 0.15 and 0.3 s.

We expected that early exposure to Chinese would be sufficient to elicit distinctive phonological sensitivity to lexical tones in international adoptees, similar to Chinese natives. This was confirmed by comparing mean MMN amplitude measured at representative electrode Fz between 0.15 and 0.3 s. The MMN elicited by the tone contrast was significantly larger than that elicited by the vowel contrast in adoptees (*t*(17) = −2.693, CI = [−1.125–0.137], *d* = −0.635, *P* = 0.046, two-tailed, Bonferroni corrected) and Chinese controls (*t*(20) = −4.994, CI = [−1.293–0.531], *d* = −1.090, *P* < 0.001, two-tailed, Bonferroni corrected), but not in Swedish controls (*t*(20) = −1.004, CI = [−0.579–0.203], *d* = −0.219, *P* = 0.98, two-tailed, Bonferroni corrected; Fig. 1*c*).

Mixed-model regression analysis (see Supplementary Tables 1 and 2) revealed interactions where the adoptees had a larger MMN amplitude difference between conditions than the Swedish controls (*β* = 0.194, SE = 0.081, *t* = 2.408, *P* = 0.018, CI = [0.034–0.354]), while the difference was smaller compared with Chinese controls (*β* = −0.168, SE = 0.081, *t* = −2.075, *P* = 0.040, CI = [−0.328–0.008]). Within-subject comparisons furthermore showed that the adoptees had longer MMN peak latency for tone than vowel trials (*t*(17) = 3.442, CI = [0.009–0.037], *d* = 0.811, *P* = 0.009, two-tailed, Bonferroni corrected), while the Swedish controls (*t*(20) = 1.627, CI = [−0.004–0.033], *d* = 0.355, *P* = 0.36, two-tailed, Bonferroni corrected) and Chinese controls (*t*(20) = −0.128, CI = [−0.018–0.016], *d* = −0.028, *P* = 1, two-tailed, Bonferroni corrected) showed no significant difference between conditions (Fig. 1*d*). An interaction between group and condition confirmed that adoptees and Chinese controls differed in their MMN latency to vowels and tones (*β* = −0.007, SE = 0.003, *t* = −2.028, *P* = 0.045, CI = [−0.013–0.000]), but this interaction was not significant when comparing adoptees and Swedish controls (*β* = 0.001, SE = 0.003, *t* = 0.350, *P* = 0.727, CI = [−0.005–0.008]).

### Vowel Sensitivity Correlates with Tone Sensitivity in Controls but Not with Age of Acquisition

To test whether tone representations established in early childhood compete with vowel representations specific to the later acquired adoptive language, we conducted a correlation analysis of deviancy effects measured for tone and vowel contrasts across individuals within each participant group. We found positive correlations between MMN amplitude for the vowel and tone contrasts for the two control groups (Chinese: *N* = 21, *r* = 0.734, *P* = 0.002, Bonferroni corrected; Swedish: *N* = 21, *r* = 0.812, *P* = 0.002, Bonferroni corrected) but not for the adoptees (*N* = 18, *r* = 0.500, *P* = 0.103, Bonferroni corrected; Fig. 1*e*). This, however, may be due to the difference in peak latency observed between the conditions. Indeed, when using peak instead of average amplitude, the correlation was significant also for the adoptees (*N* = 18, *r* = 0.682, *P* = 0.005, Bonferroni corrected). Thus, as MMN modulation elicited by the vowel contrast increased, the MMN modulation elicited by the tone contrast also increased.

We also found a positive correlation between the peak latencies of MMN elicited by vowel and tone contrasts in the adoptees (*N* = 18, *r* = 0.598, *P* = 0.027, Bonferroni corrected), but the same tests did not reach significance in the control groups (Swedish: *N* = 21, *r* = 0.366, *P* = 0.306, Bonferroni corrected; Chinese: *N* = 21, *r* = 0.156, *P* = 1, Bonferroni corrected; Fig. 1*f*). There was, however, no correlation in adoptees between mean MMN amplitude and age of adoption, either for the tone contrast (*N* = 18, *r* = −0.051, *P* = 0.842) or the vowel contrast (*N* = 18, *r* = −0.226, *P* = 0.366), suggesting that the duration of exposure to the native contrast did not relate to phonological sensitivity in adulthood.

### Tone Contrast Elicits Increased Theta and Alpha Power in Adoptees

We also computed spectral power and phase locking differences across trials in order to further characterize the oscillatory patterns underlying scalp recorded MMN deflections (Makeig et al. 2004). We expected that true MMN responses to phonological deviants would manifest as increased synchronization and phase alignment in the theta range, indexing underlying memory comparison processes (Fuentemilla et al. 2008; Sauseng and Klimesch 2008; Bishop et al. 2011).

**Figure 2:**
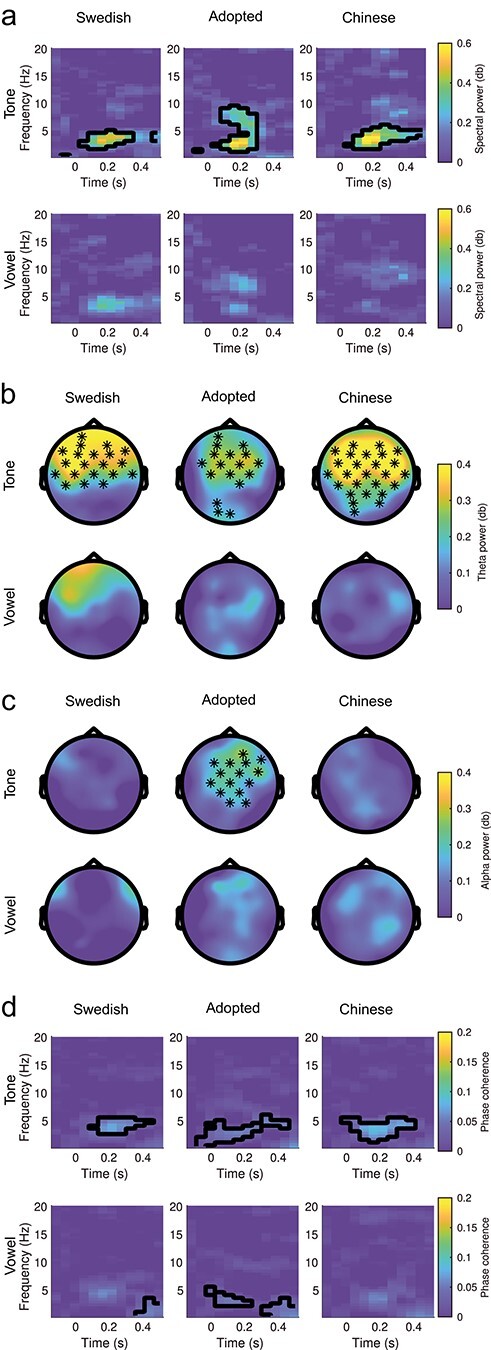
MMN spectral power and phase coherence results. (*a*) Deviant minus standard power spectrum intensity over time at electrode Fz for tone (top) and vowel (bottom) deviants in the three experimental groups with significant clusters outlined. Differences were limited to the lower theta range in Chinese controls (*t*_sum_ = 3293.72, SD = 0.002, CI_range_ = 0.004, *P* = 0.004) and Swedish controls (*t*_sum_ = 2452.46, SD = 0.002, CI_range_ = 0.004, *P* = 0.005) but extended higher in the alpha range for tones for adoptees (*t*_sum_ = 22.844, SD = 0.002, CI_range_ = 0.004, *P* = 0.004). (*b*) Spectral power topoplots (deviant minus standard) at Fz between 0.15 and 0.3 s after stimulus onset time in the theta (3–5 Hz) range. Electrodes showing significant differences detected by cluster-based permutation tests are indicated by a black dot (*P* < 0.05). (*c*) Topoplots in the alpha (7–9 Hz) range. Differences only occurred for the tone contrast in adoptees. (*d*) Phase coherence over time at electrode Fz with significant deviant versus standard differences outlined for adoptees (*t*_sum_ = 2127.02, SD = 0.001, CI_range_ = 0.002, *P* = 0.001), Swedish controls (*t*_sum_ = 365.352, SD = 0.002, CI_range_ = 0.005, *P* = 0.006), and Chinese controls (*t*_sum_ = 2370.60, SD = 0.001, CI_range_ = 0.003, *P* = 0.002) in the tone condition, and in the vowel condition MMN time window for the adoptees (*t*_sum_ = 623.16, SD = 0.005, CI_range_ = 0.009, *P* = 0.023) and for adoptees (*t*_sum_ = 1042.42, SD = 0.003, CI_range_ = 0.006, *P* = 0.009) and Swedish natives (*t*_sum_ = 789.61, SD = 0.006, CI_range_ = 0.012, *P* = 0.036) in the phonological categorization time window.

Cluster-based permutation tests (Maris and Oostenveld 2007) over all electrodes, samples, and frequencies revealed increased theta synchronization for the tone stimulus compared with the standard stimulus in the MMN time window (Chinese controls: 2–6.5 Hz, cluster *P* = 0.004, 0.1–0.38 s, two-tailed; Adoptees: 2.5–10 Hz, cluster *P* = 0.01, 0.1–0.48 s, two-tailed, Swedish controls: 2.5–4.5 Hz, 0.1–0.54 s, cluster *P* = 0.005, two-tailed; Fig. 2*a*). This was observed over frontal scalp locations in all groups (Fig. 2*b*). However, only in the adoptees did spectral power increases extend into the alpha range (up to 10 Hz) over frontal electrodes in the early part of the time window (cluster *P* = 0.002, Fig. 2*c*), hinting at the fact that additional processes were at play in this group. We found no significant synchronization clusters for the vowel contrast in any of the groups or any of the frequency bands surveyed (*P*s > 0.12).

Finally, we compared intertrial coherence between the standard and the deviant conditions as a measure of stimulus phase-locking across trials (Delorme and Makeig 2004), corresponding to the “phase-locking factor” (Tallon-Baudry et al. 1996). Once again, we found significant theta clusters in the tone condition for all groups in the MMN time window at frontal sites (Adoptees: 2–4.5 Hz, 0–0.43 s, *P* < 0.001, two-tailed; Chinese: 1.5–5.5 Hz, 0–0.38 s, *P* = 0.002; Swedish: 3–5.5 Hz, 0.1–0.43 s, *P* = 0.009, Fig. 2*d*). In the vowel condition, there were early increases in coherence in the theta range in the case of adoptees (2.5–6 Hz, 0–0.21 s, *P* = 0.023) and that of Swedish natives (4–6 Hz, 0.05–0.27 s, *P* = 0.068, two-tailed) although the cluster was only marginally significant in the latter group. In addition, there were significant clusters in a later time window for both adoptees (0.5–3.5 Hz, 0.32–0.54 s, *P* = 0.009, two-tailed) and Swedish natives (0.5–3.5 Hz, 0.38–0.59 s, *P* = 0.036, two-tailed), which did not reach significance in the Chinese native group (0.5–2 Hz, 0.32–0.48 s, *P* = 0.09, two-tailed).

## Discussion

While it is widely acknowledged that early language experience is critical for language development, the extent to which early acquired phonological contrasts can be lost and the extent to which new contrasts can be acquired after adoption remain largely unknown. The international adoptees tested here were born in China and exposed to Chinese tonal contrasts within their first two years of life (mean = 18 months), before being adopted by Swedish families. Despite having had essentially no re-exposure to Chinese for over 15 years and having not been trained prior to the experimental session, these adoptees displayed a striking increase of MMN amplitudes elicited by native tone contrasts of Mandarin Chinese compared with adoptive Swedish vowel contrasts, similar to that observed in Chinese native controls. Meanwhile, the relative MMN increase in adoptees was not observed in native Swedish controls.

### M‌MN Amplitude Reflects Retained Neural Specialization for the Native Language

MMN amplitude modulation provides evidence of pre-attentive phonological sensitivity to the native language (Näätänen et al. 1997). While significant MMN modulations were found for both phonological contrasts in all groups—showing that differences between groups thus cannot be attributed to a lack of sensitivity of our index of choice—responses elicited by the tonal contrast were larger than those elicited by the vowel contrast in adoptees and Chinese controls, but not in Swedish controls. Native phonological representations thus appear to be highly resilient over time in adoptees.

The results are consistent with previous neuroimaging findings on first and second language acquisition in international adoptees. For instance, previous studies have shown similar activation levels in the left temporal cortex of adoptees and Chinese–French bilinguals, but not in nonnative controls, in response to a native Chinese tone contrast (Pierce et al. 2014). However, because traces of the native language in adoptees are often assumed to have become dormant (Hyltenstam et al. 2009; Oh et al. 2009), previous studies have involved a period of practice prior to the experimental task in order to familiarize participants with the contrasts used (e.g., Pierce et al. 2014) or have relied on behavioral discrimination improvement over time as the outcome measure (Singh et al. 2011; Choi et al. 2017). Yet, here, we detected sensitivity to the tonal contrast in adoptees who had not undergone any training or pre-exposure to the phonological contrast ahead of testing. This indicates that irreversible auditory sensitivities established during the optimal period for speech development have long-lasting effects on the neural patterns of acoustic processing, possibly for life.

### Inhibitory Control Regulates Native Contrast Sensitivity in Adoptees

To further characterize amplitude and latency differences between adoptees and controls, we examined the oscillatory patterns underlying the MMN. While all groups showed increased theta band synchronization in response to Chinese lexical tone deviants—reflecting the acoustically and perceptually salient nature of the fundamental pitch difference—the adoptees showed additional spectral power increases in the alpha range. On the one hand, theta range power perturbations in an MMN paradigm have previously been interpreted as indexing memory comparison processes (Fuentemilla et al. 2008). On the other hand, alpha range spectral power increases have been associated with the engagement of attention and monitoring (Klimesch 1999), as well as inhibitory control (Knyazev 2007). Given that we found clear signs of retained neural specialization for the native pitch contrast in the adoptees, the neural population responsive to such contrasts is likely activated by spurious tonal variations in daily Swedish input (especially considering that such variations have more restricted functional significance in Swedish). This would lead the adoptees’ language system to constantly wander off the main track of Swedish phonology. It makes sense that adoptees would resort to additional cognitive strategies, be it through increased attention or inhibitory control, or both, in order to discard the processing noise arising from nonnative contrasts while displaying native-like comprehension of the adoptive language. For instance, inhibitory control could be applied to neural populations involved in the perception of fundamental frequency to attenuate their sensitivity to pitch modulations in the speech signal (Strauß et al. 2014). In line with previous evidence for top-down modulation involving alpha synchronization at prefrontal sites (Sauseng et al. 2005), we interpret our results as reflecting a mechanism of top-down inhibition indexed by increases in alpha power. This provides an account for the relative delay in MMN peak latency for the tone contrast observed between adoptees and Chinese controls. Such processes may not be detectable with fMRI due to the slow evolution of the BOLD response, and given that excitation and inhibition mechanisms can result in highly similar activations in fMRI (Logothetis 2008). Admittedly, functional evidence for cognitive control involvement was obtained by Pierce et al. (2015) in both adoptees and bilingual Chinese controls engaged in a phonological working memory task featuring adoptive language phonemes, consistent with long-term compensatory effects from early language specialization. While the cognitive control mechanisms engaged were not specified by Pierce et al. (2015), here, we propose that the cognitive mechanisms involved entail inhibitory control.

Furthermore, increased theta band phase coherence, thought to underlie MMN generation (Fuentemilla et al. 2008; Bishop and Hardiman 2010; Lakatos et al. 2020), was observed in all groups for the tone contrast, but only in the adoptees and Swedish natives for the vowel contrast. One reason why the response pattern elicited by the native and nonnative contrasts was not inverted between control groups is that, although combining different acoustical features within the same oddball task is common practice (Näätänen et al. 2004), presenting a fundamental frequency and spectral formant modulation concurrently may have inhibited the response to the acoustically and perceptually less salient formant deviation (Bishop and Hardiman 2010). Yet, phase coherence increase in adoptees and Swedish natives was also observed in a later time window associated with phonological categorization of vowels (Hill et al. 2004) suggesting that the vowel contrast did elicit phonological processes in both Swedish speaking groups, albeit beyond the canonical MMN range.

Long-term experience with an early acquired second language may thus have led adoptees to recruit the same processing mechanisms for a Swedish vowel contrast as that recruited by Swedish natives, while at the same time actively attenuating neural responses to native language pitch variations, suggesting the coexistence of plasticity and stability of early learning.

### Adaptation in Adulthood Is Not Singly Explained by Interference or Maturation

The current findings contribute to a long-standing theoretical debate in the study of language development regarding the nature of early and later language learning. Retention of native language sensitivity in international adoptees has been suggested to interfere with the acquisition of the adoptive language, with the loss of native language sensitivity sometimes considered a prerequisite for native-like language attainment (Pallier et al. 2003). If such was the case, however, the degree to which the native language is retained in adoptees could be expected to correlate negatively with the processing of phonological contrasts in the adoptive language (Bylund et al. 2012). To our knowledge, however, the proposal has not been previously tested directly in international adoptees. While some studies have found a negative correlation between measurements of pronunciation in the first and second languages of bilingual speakers, these results have been interpreted as interference relating to the continuous use of two languages (Yeni-Komshian et al. 2000), an explanation which is not viable for international adoptees. Here, for the first time, our data provide a picture of the way in which the native and adopted languages interact at the neural level over time. Instead of negative correlations between responses to tone and vowel contrasts, we found positive correlations between MMN amplitude elicited by tone and vowel contrasts in all participant groups. This not only suggests that a highly sensitive phonological system supports phonological processing of both native and foreign contrasts alike but also that the maintenance of native language sensitivity does not significantly impede the acquisition of the adoptive language. Furthermore, if brain maturation had played a critical role, we would expect stabilization of early language representations over time, and decreased plasticity in adoptive language acquisition, leading to positive correlations for the native contrast and negative correlations for the adoptive contrast. The responses, however, did not correlate with the age of acquisition, suggesting that the effect may not be strictly maturational in origin.

Previously, the question of age of language acquisition has been studied primarily through bilingual and deaf populations. Delayed acquisition in adoptees is, however, not contingent on congenital hearing loss and learning of the second language is not confounded with bilingual language use. Instead, through the experience of sequential monolingual language acquisition, adoptees provide a better gauge of the influence of age on language acquisition.

## Conclusion

Our findings provide compelling evidence for a profound and durable shaping of the perceptual system by early language experience. Taken together, event-related potential data and time–frequency analyses place the adoptees in a unique relation to speakers of both the native and the adoptive language. On the one hand, they retain neural sensitivity to a forgotten native Chinese lexical tone contrast, a responsiveness which may need to be suppressed following adoption, likely through inhibitory control mechanisms. On the other hand, they show only slightly attenuated neural sensitivity to the adoptive Swedish vowel contrast and are fully fluent speakers of the language. Irreversible specialization for the native language may thus be accommodated by flexible recruitment of additional cognitive resources. This is reflected in the concurrent inhibition of responses to the native contrast, and distinguishable nonnative processing patterns of the adoptive language contrast. Such long-term influences of early experience on language processing in adulthood may stem from the establishment of structural constraints on stimulus-specific processing networks in the brain (Takesian and Hensch 2013) as well as through the recruitment of differential mechanisms to accommodate nonnative linguistic exposure. In either case, the question is not so much whether the system underpinning phonological discrimination is forever set in early childhood (i.e., a rigid critical period account) or entirely reconfigurable through neuroplasticity throughout life, but rather how the same neurophysiological system can adapt to different sets of perceptual characteristics and constraints introduced in succession.

## Funding

Lars Hierta Memorial Foundation to G.N.; IDO Foundation to G.N.; National Research Foundation (Grant IFR180306315940 to E.B.); European Research Council (Grant ERC-209704 to G.T.); Economic and Social Research Council Centre for Research on Bilingualism in Theory and Practice, Bangor University (Grant ES/E024556/1); GT is supported by the Polish National Agency for Academic Exchange (NAWA) under the NAWA Chair Programme (PPN/PRO/2020/1/00006).

## Notes

We would like to thank Janet Werker, Marilyn Vihman, Dominik Freunberger, and three anonymous reviewers for valuable comments on earlier versions of the paper. *Conflict of Interest*: Authors declare no conflicts of interest.

## Supplementary Material

Irreversible_Specialization_Supplementary_bhab447

## References

[ref1] Bishop DV , Hardiman MJ, Barry JG. 2011. Is auditory discrimination mature by middle childhood? A study using time-frequency analysis of mismatch responses from 7 years to adulthood. Dev Sci. 14:402–416. 22213909 10.1111/j.1467-7687.2010.00990.x PMC3083517

[ref2] Bishop DVM , Hardiman MJ. 2010. Measurement of mismatch negativity in individuals: a study using single-trial analysis. Psychophysiology. 47:697–705. 20210877 10.1111/j.1469-8986.2009.00970.x PMC2904495

[ref3] Bowers JS , Mattys SL, Gage SH. 2009. Preserved implicit knowledge of a forgotten childhood language. Psychol Sci. 20:1064–1069. 19645694 10.1111/j.1467-9280.2009.02407.x

[ref4] Bylund E , Abrahamsson N, Hyltenstam K. 2012. Does first language maintenance hamper nativelikeness in a second language? Stud Second Lang Acquis. 34:215–241.

[ref5] Cheour M , Ceponiene R, Lehtokoski A, Luuk A, Allik J, Alho K, Näätänen R. 1998. Development of language-specific phoneme representations in the infant brain. Nat Neurosci. 1:351–353. 10196522 10.1038/1561

[ref6] Choi J , Broersma M, Cutler A. 2017. Early phonology revealed by international adoptees’ birth language retention. Proc Natl Acad Sci. 114:7307–7312. 28652342 10.1073/pnas.1706405114 PMC5514759

[ref7] Delorme A , Makeig S. 2004. EEGLAB: an open source toolbox for analysis of single-trial EEG dynamics including independent component analysis. J Neurosci Methods. 134:9–21. 15102499 10.1016/j.jneumeth.2003.10.009

[ref8] Duanmu S . 2007. Phonology of standard Chinese. Oxford: Oxford University Press.

[ref9] Friederici AD , Steinhauer K, Pfeifer E. 2002. Brain signatures of artificial language processing: evidence challenging the critical period hypothesis. Proc Natl Acad Sci. 99:529–534. 11773629 10.1073/pnas.012611199 PMC117594

[ref10] Fuentemilla L , Marco-Pallarés J, Münte TF, Grau C. 2008. Theta EEG oscillatory activity and auditory change detection. Brain Res. 1220:93–101. 18076870 10.1016/j.brainres.2007.07.079

[ref11] Hernandez A , Li P, MacWhinney B. 2005. The emergence of competing modules in bilingualism. Trends Cogn Sci. 9:220–225. 15866148 10.1016/j.tics.2005.03.003 PMC4107303

[ref12] Hill PR , McArthur GM, Bishop DVM. 2004. Phonological categorization of vowels: a mismatch negativity study. Neuroreport. 15:2195–2199. 15371732 10.1097/00001756-200410050-00010

[ref13] Hyltenstam K , Bylund E, Abrahamsson N, Park H-S. 2009. Dominant-language replacement: the case of international adoptees. Biling Lang Congn. 12:121–140.

[ref14] Klimesch W . 1999. EEG alpha and theta oscillations reflect cognitive and memory performance: a review and analysis. Brain Res Rev. 29:169–195. 10209231 10.1016/s0165-0173(98)00056-3

[ref15] Knyazev GG . 2007. Motivation, emotion, and their inhibitory control mirrored in brain oscillations. Neurosci Biobehav Rev. 31:377–395. 17145079 10.1016/j.neubiorev.2006.10.004

[ref16] Kuhl PK . 2004. Early language acquisition: cracking the speech code. Nat Rev Neurosci. 5:831–843. 15496861 10.1038/nrn1533

[ref17] Kuhl PK , Williams KA, Lacerda F, Stevens KN, Lindblom B. 1992. Linguistic experience alters phonetic perception in infants by 6 months of age. Science. 255:606–608. 1736364 10.1126/science.1736364

[ref18] Lakatos P , O’Connell MN, Barczak A, McGinnis T, Neymotin S, Schroeder CE, Smiley JF, Javitt DC. 2020. The Thalamocortical circuit of auditory mismatch negativity. Biol Psychiatry. 87:770–780. 31924325 10.1016/j.biopsych.2019.10.029 PMC7103554

[ref19] Logothetis NK . 2008. What we can do and what we cannot do with fMRI. Nature. 453:870–878. 10.1038/nature06976 18548064

[ref20] Makeig S , Debener S, Onton J, Delorme A. 2004. Mining event-related brain dynamics. Trends Cogn Sci. 8:204–210. 15120678 10.1016/j.tics.2004.03.008

[ref21] Maris E , Oostenveld R. 2007. Nonparametric statistical testing of EEG- and MEG-data. J Neurosci Methods. 164:177–190. 17517438 10.1016/j.jneumeth.2007.03.024

[ref22] Näätänen R . 1995. The mismatch negativity: a powerful tool for cognitive neuroscience. Ear Hear. 16:6–18. 7774770

[ref23] Näätänen R , Lehtokoski A, Lennes M, Cheour M, Huotilainen M, Iivonen A, Vainio M, Alku P, Ilmoniemi RJ, Luuk A, et al. 1997. Language-specific phoneme representations revealed by electric and magnetic brain responses. Nature. 385:432–434. 9009189 10.1038/385432a0

[ref24] Näätänen R , Pakarinen S, Rinne T, Takegata R. 2004. The mismatch negativity (MMN): towards the optimal paradigm. Clin Neurophysiol. 115:140–144. 14706481 10.1016/j.clinph.2003.04.001

[ref25] Näätänen R , Picton T. 1987. The N1 wave of the human electric and magnetic response to sound: a review and an analysis of the component structure. Psychophysiology. 24:375–425. 3615753 10.1111/j.1469-8986.1987.tb00311.x

[ref26] Oh JS , Au TK-F, Jun S-A. 2009. Early childhood language memory in the speech perception of international adoptees. J Child Lang. 37:1123–1132. 19951452 10.1017/S0305000909990286

[ref27] Oldfield RC . 1971. The assessment and analysis of handedness: the Edinburgh inventory. Neuropsychologia. 9:97–113. 5146491 10.1016/0028-3932(71)90067-4

[ref28] Oostenveld R , Fries P, Maris E, Schoffelen J-M. 2011. FieldTrip: open source software for advanced analysis of MEG, EEG, and invasive electrophysiological data. Comput Intell Neurosci. 2011:1–9. 21253357 10.1155/2011/156869 PMC3021840

[ref29] Pallier C , Dehaene S, Poline J-B, LeBihan D, Argenti A-M, Dupoux E, Mehler J. 2003. Brain imaging of language plasticity in adopted adults: can a second language replace the first? Cereb Cortex. 13:155–161. 12507946 10.1093/cercor/13.2.155

[ref30] Pierce LJ , Chen J-K, Delcenserie A, Genesee F, Klein D. 2015. Past experience shapes ongoing neural patterns for language. Nat Commun. 6:10073. 26624517 10.1038/ncomms10073 PMC4686754

[ref31] Pierce LJ , Klein D, Chen J-K, Delcenserie A, Genesee F. 2014. Mapping the unconscious maintenance of a lost first language. Proc Natl Acad Sci. 111:17314–17319. 25404336 10.1073/pnas.1409411111 PMC4260567

[ref32] Reh RK , Dias BG, Nelson CA, Kaufer D, Werker JF, Kolb B, Levine JD, Hensch TK. 2020. Critical period regulation across multiple timescales. Proc Natl Acad Sci. 117:23242–23251. 32503914 10.1073/pnas.1820836117 PMC7519216

[ref33] Riad T . 2014. The phonology of Swedish, The phonology of the world’s languages. Oxford, UK: Oxford University Press.

[ref34] Rivera-Gaxiola M , Silva-Pereyra J, Kuhl PK. 2005. Brain potentials to native and non-native speech contrasts in 7- and 11-month-old American infants. Dev Sci. 8:162–172. 15720374 10.1111/j.1467-7687.2005.00403.x

[ref35] Sassenhagen J , Draschkow D. 2019. Cluster-based permutation tests of MEG/EEG data do not establish significance of effect latency or location. Psychophysiology. 56:e13335. https://www.draschkow.com/app/download/9767211/16267843.pdf. 30657176 10.1111/psyp.13335

[ref36] Sauseng P , Klimesch W. 2008. What does phase information of oscillatory brain activity tell us about cognitive processes? Neurosci Biobehav Rev. 32:1001–1013. 18499256 10.1016/j.neubiorev.2008.03.014

[ref37] Sauseng P , Klimesch W, Doppelmayr M, Pecherstorfer T, Freunberger R, Hanslmayr S. 2005. EEG alpha synchronization and functional coupling during top-down processing in a working memory task. Hum Brain Mapp. 26:148–155. 15929084 10.1002/hbm.20150 PMC6871735

[ref38] Sharbrough F , Chatrian G, Lesser R, Luders H, Nuwer M, Picton T. 1991. American electroencephalographic society guidelines for standard electrode position nomenclature. Clin Neurophysiol. 8:200–202. 2050819

[ref39] Singh L , Liederman J, Mierzejewski R, Barnes J. 2011. Rapid reacquisition of native phoneme contrasts after disuse: you do not always lose what you do not use. Dev Sci. 14:949–959. 21884311 10.1111/j.1467-7687.2011.01044.x

[ref40] Strauß A , Wöstmann M, Obleser J. 2014. Cortical alpha oscillations as a tool for auditory selective inhibition. Front Hum Neurosci. 8:1–7. 24904385 10.3389/fnhum.2014.00350 PMC4035601

[ref41] Takesian AE , Hensch TK. 2013. Balancing plasticity/stability across brain development. In: Merzenich MM, Nahum M, Van Vleet TM, editors. Progress in brain research. Changing Brains: Elsevier, pp. 3–34. 10.1016/B978-0-444-63327-9.00001-1 24309249

[ref42] Tallon-Baudry C , Bertrand O, Delpuech C, Pernier J. 1996. Stimulus specificity of phase-locked and non-phase-locked 40 Hz visual responses in human. J Neurosci. 16:4240–4249. 8753885 10.1523/JNEUROSCI.16-13-04240.1996 PMC6579008

[ref43] Tees RC , Werker JF. 1984. Perceptual flexibility: maintenance or recovery of the ability to discriminate non-native speech sounds. Can J Psychol. 38:579–590. 6518419 10.1037/h0080868

[ref44] Tsao F-M , Liu H-M, Kuhl PK. 2004. Speech perception in infancy predicts language development in the second year of life: a longitudinal study. Child Dev. 75:1067–1084. 15260865 10.1111/j.1467-8624.2004.00726.x

[ref45] Uylings HBM . 2006. Development of the human cortex and the concept of “critical” or “sensitive” periods. Lang Learn. 56:59–90.

[ref46] Ventureyra VAG , Pallier C, Yoo H-Y. 2004. The loss of first language phonetic perception in adopted Koreans. J Neurolinguistics. 17:79–91.

[ref47] Werker JF , Hensch TK. 2015. Critical periods in speech perception: new directions. Annu Rev Psychol. 66:173–196. 25251488 10.1146/annurev-psych-010814-015104

[ref48] Werker JF , Tees RC. 2005. Speech perception as a window for understanding plasticity and commitment in language systems of the brain. Dev Psychobiol. 46:233–251. 15772961 10.1002/dev.20060

[ref49] Yeni-Komshian GH , Flege JE, Liu S. 2000. Pronunciation proficiency in the first and second languages of Korean-English bilinguals. Biling Lang Congn. 3:131–149.

