# Correction to: Irreversible specialization for speech perception in early international adoptees

**DOI:** 10.1093/cercor/bhac181

**Published:** 2022-04-25

This is a correction to: Gunnar Norrman, Emanuel Bylund, Guillaume Thierry, Irreversible Specialization for Speech Perception in Early International Adoptees, *Cerebral Cortex*, 2021, bhab447, https://doi.org/10.1093/cercor/bhab447.

In the originally published version of this manuscript, the following funding statement was omitted from the Funding section:

“GT is supported by the Polish National Agency for Academic Exchange (NAWA) under the NAWA Chair Programme (PPN/PRO/2020/1/00006)”

This error has been corrected.

