# Supplementary material for: Irreversible specialization for speech perception in early international adoptees
Source: Cereb Cortex. 2021 Dec 25;32(17):3777–85. doi: 10.1093/cercor/bhab447 (PMC9433419; doi:10.1093/cercor/bhab447)
Supplement: Irreversible_Specialization_Supplementary_bhab447 [file irreversible_specialization_supplementary_bhab447.pdf]

Irreversible specialization for speech perception in early international adoptees – Supplemental information

Table 1: Model coefficients for the MMN amplitude analysis. The model included the effect coded categorical variables group (adopted, Swedish, Chinese) and condition (tone, vowel), with current age, gender, and handedness as covariates. The variable age was centered around zero and scaled to a unit standard deviation of 1. Handedness was assessed using the Edinburgh Handedness inventory, and values above 0 were treated as right handed. Random effects included by-participant random intercept. Average MMN amplitude at channel Fz between 0.15–0.3 s for each trial was used as the dependent variable.

Name	Estimate	SE	t	DF	p	Lower	Upper
(Intercept)	-0.84	1.335	-0.629	111	0.531	-3.486	1.806
Group Swedish	0.162	0.211	0.768	111	0.444	-0.256	0.58
Group Chinese	-0.218	0.23	-0.948	111	0.345	-0.674	0.238
Female	-0.041	0.052	-0.787	111	0.433	-0.144	0.062
Condition Tone	-0.289	0.058	-4.987	111	<0.001	-0.403	-0.174
Handedness	-0.043	0.832	-0.051	111	0.959	-1.69	1.605
Age	0.01	0.164	0.060	111	0.952	-0.314	0.334
Group Swedish:Condition Tone	0.194	0.081	2.408	111	0.018	0.034	0.354
Group Chinese:Condition Tone	-0.168	0.081	-2.075	111	0.040	-0.328	-0.008

Table 2: Model coefficients for the MMN latency analysis. The effect coded variables group and condition, and the covariates gender, age, and handedness, were used as fixed effects, with by-participant random intercept. The variable age was centered around zero and scaled to a unit standard deviation of 1. Handedness was assessed using the Edinburgh Handedness inventory, and values above 0 were treated as right handed. The outcome variable was the peak latency between 0.15–0.3 s in the deviant-minus-standard difference wave.

Name	Estimate	SE	t	DF	p	Lower	Upper
(Intercept)	0.266	0.02	13.04	111	<0.001	0.226	0.307
Group Swedish	-0.006	0.005	-1.219	111	0.226	-0.016	0.004
Group Chinese	0.002	0.005	0.294	111	0.769	-0.009	0.012
Female	0.007	0.008	0.811	111	0.419	-0.009	0.023
Condition Tone	0.006	0.002	2.609	111	0.010	0.001	0.011
Handedness	-0.043	0.019	-2.232	111	0.028	-0.081	-0.005
Age	0.002	0.004	0.556	111	0.579	-0.005	0.009
Group Swedish:Condition Tone	0.001	0.003	0.350	111	0.727	-0.005	0.008
Group Chinese:Condition Tone	-0.007	0.003	-2.028	111	0.045	-0.013	0.001

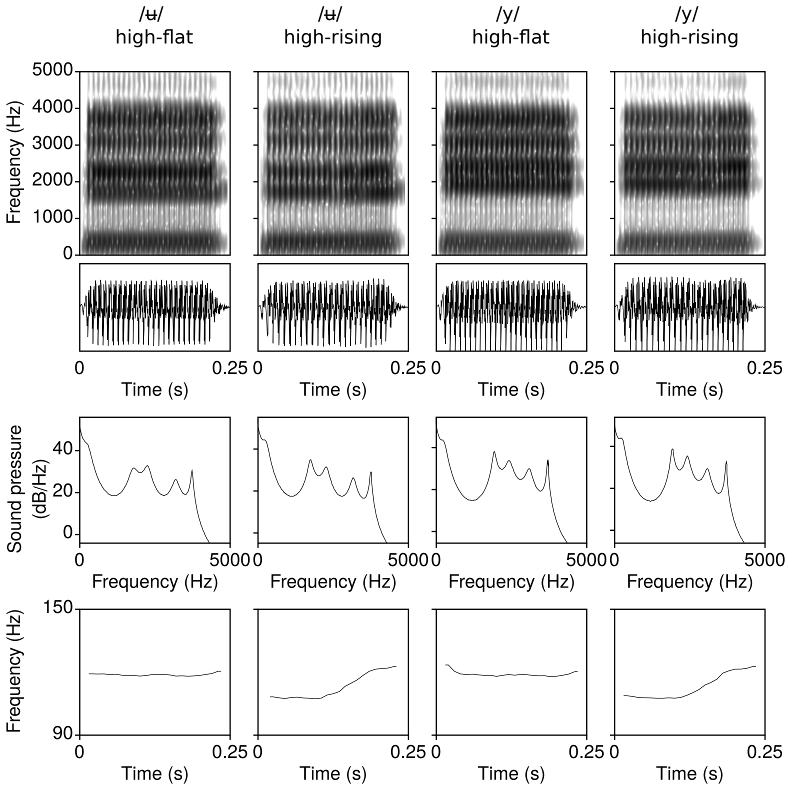

Figure 1: Auditory stimuli used in the experiment. Each panel displays the spectrogram, amplitude, spectrum (LPC smoothed, pre-emphasis 50 Hz), and pitch envelope, for combined Swedish vowels (/y/, /ʉ/) and Chinese lexical tones (high-flat, high-rising).
